# 

**Published:** 1888-01

**Authors:** 


WHOLE NUMBER, CCCXVIII.

JANUARY, 1888.

NUMBER 6. VOLUME XXVII.

### THE BUFFALO Medical AND Surgical Journal

ESTABLISHED YEAR 1844.

LIBRARY INDEXED S.G.O.

**EDITORS:**

**THOS. LOTHROP, M. D.** **A. R. DAVIDSON, M. D.**

**ASSOCIATE EDITORS:**

FLOYD S. CREGO, M. D. CARLTON C. FREDERICK, M. D.
FRED. R. CAMPBELL, M. D. FRANK H. POTTER, M. D.

EDWARD B. ANGELL, M. D., Rochester, N. Y.

Entered at the Post-Office at Buffalo, N. Y., as Second-class Mail Matter.

